# Phenolic and Antioxidant Compound Accumulation of *Quercus robur* Bark Diverges Based on Tree Genotype, Phenology and Extraction Method

**DOI:** 10.3390/life13030710

**Published:** 2023-03-06

**Authors:** Vaida Sirgedaitė-Šėžienė, Ieva Čėsnienė, Gabija Leleikaitė, Virgilijus Baliuckas, Dorotėja Vaitiekūnaitė

**Affiliations:** Institute of Forestry, Lithuanian Research Centre for Agriculture and Forestry, Liepų Str. 1, LT-53101 Girionys, Lithuania

**Keywords:** ABTS, DPPH, early bud burst, English oak, half-sib, late bud burst, pedunculate oak, total flavonoid content, total phenol content, natural products

## Abstract

Oak bark is a rich niche for beneficial bioactive compounds. It is known that the amount of the compounds found in plant tissues can depend on species, genotype, growth site, etc., but it is unclear whether oak phenology, i.e., late or early bud burst, can also influence the amount of phenols and antioxidants that can be extracted. We tested two *Quercus robur* populations expressing different phenology and five half-sib families in each population to see how phenology, genotype, as well as extrahent differences (75% methanol or water) can determine the total phenol, total flavonoid content, as well as antioxidant activity. Significant statistical differences were found between half-sib families of the same population, between populations representing different oak phenology and different extrahents used. We determined that the extraction of flavonoids was more favorable when using water. So was antioxidant activity using one of the indicators, when significant differences between extrahents were observed. Furthermore, in families where there was a significant difference, phenols showed better results when using methanol. Overall, late bud burst families exhibited higher levels in all parameters tested. Thus, we recommend that for further bioactive compound extraction, all these factors be noted.

## 1. Introduction

In recent years, there has been a lot of interest in functional compounds derived from plants and also notably from byproducts of plant-associated industries, as a way to reduce waste and provide additional valorization [1,2]. For example, wine-making and leather tanning enterprises have used oaks (*Quercus*) for centuries. It is known that specific compounds from trees in the oak genus facilitate taste in aged alcoholic drinks and are responsible for altering the protein structure in the hides to induce durability [3,4]. However, research shows that oaks contain many useful compounds that may be utilized in multiple ways in varied other fields, i.e., as medicine, supplements, and additives. Oak flour has been used for centuries and recently has been reintroduced as a bioactive ingredient in human foodstuffs [5]. Furthermore, Gamboa-Gomez et al. demonstrated that oak leaf infusions can be used as additives that could potentially be anti-hyperglycemic and have antioxidative effects in mice [6]. Oak leaf extracts were also shown to reduce lipid oxidation, increase antioxidant capacity and reduce bacterial growth in meat [7,8]. Moreover, oak leaf extracts were shown to modify rumen fermentation, thus alleviating the oxidative imbalance ruminant animals face [9]. As an additive to common carp fish food, oak leaf extracts were shown to stimulate antioxidant and immune system of the carp and reduce stress [10].

Thus, as can be seen, oak tissues contain many beneficial compounds; however, more and more research is geared toward compounds that could be located in oak bark specifically, as it has been singled out as a potential byproduct for valorization [11,12,13,14]. Mirski et al. showed that ground oak bark can be used as a filler in plywood adhesives [11]. Furthermore, oak bark-based animal feed additives were reported to positively affect chicken immunity [15]. Moreover, oak bark-derived tannins were suggested for use in medicinal topical creams meant for allergy treatment [16]. Oak bark extracts were also shown to work in treating periodontal disease [17]. Additionally, multiple studies reported that oak bark derivatives were demonstrated to have antibacterial properties [13,18,19,20]. Similarly, as with oak leaves, oak bark derivatives were reported to positively affect digestion in the rumen [21]. Oak bark compounds were also reported to have been successfully used as an effective additive in yogurt production [22]. 

Oaks are rich in phenolic compounds [1,20], that can correlate positively with antioxidative capacity [23]. Several studies report on the composition of oak bark extracts. Elansary et al. found high levels of antioxidant phenols such as ellagic, gallic, protocatechuic, vanillic and caffeic acids and catechin derivatives in oak bark. Perhaps most notably, high levels of ellagic acid were reported [24]. Recently ellagic acid has been linked with multiple health benefits and as such is a prized bioactive compound [25]. Complimentary to Elansary et al., Ucar and Ucar also report on catechin and ellagic acid [24,26]. Ucar and Ucar also observed sitosterol and quercitol as notable oak bark derivatives [26]. While at the moment research into the direct effects these compounds may have on human health are scarce, preliminary data suggest that catechin, sitosterol, and quercitol may reduce blood pressure [27], have antidiabetic properties [28], and have cholesterol-lowering properties [29], respectively. Furthermore, a 2015 study refers to the anti-quorum sensing and antimicrobial capabilities of oak bark extracts and identified at least two compounds that were responsible—1,2,3-benzenetriol and 4-propyl-1,3-benzenediol [30]. Since this field is not well researched, it is possible to find even more of these beneficial bioactive compounds in oak bark. 

It has been observed that the amount of varied compounds within plant tissues are potentially determined by a multitude of factors, such as genotype [31,32], species [4,33], leaf age and seasonal variations [34,35], growth site [35], stress [36], and extraction methodology [13,14,20,37].

Oaks can have phenotype differences, i.e., they may be late or early in terms of bud burst/flushing. This is a natural mechanism that may help protect early bud burst oak trees from herbivore or pathogen attacks and also protect late bud burst oaks from frost damage as well as certain herbivore attacks [38,39,40]. 

Since phenolic compounds and antioxidants are involved in protection from both biotic and abiotic stressors [36,41], it is worth investigating whether either early or late bud burst oak phenotypes may produce larger amounts of them in their tissues and thus the bark of oaks with this phenotype would potentially be more beneficial for extracting bioactive compounds of phenolic or antioxidant origin.

Based on all these data, in order to optimize the process of extracting bioactive compounds from oak bark, it is important to accurately determine all the possible factors that may impact polyphenol or antioxidant production. We hypothesized that *Quercus robur* (pedunculate oak) bark phenolics and antioxidant activity levels were determined not only by genotype (different half-sib families) but also by tree phenology (early and late bud flushing/burst time). We also looked into the variation that could be introduced in this process due to the use of different extrahents, i.e., methanol and distilled water. This may be important, as some extracted compounds may be used for foodstuffs and thus methanol may not be appropriate due to health or other concerns. 

We found significant differences in phenolics and antioxidant extraction efficiency from oak bark between half-sib families of the same population, between populations representing different oak phenology, and different extrahents used.

## 2. Materials and Methods

### 2.1. Test Subjects and Tree Phenology Evaluation

Ten half-sib families from two *Q. robur* populations—Josvainiai and Dūkštos—(five from each) were studied when trees were 24 years old. All trees were growing in the same growth site. The stage of budburst was recorded and a value from 0 to 6 was assigned, with the larger figure corresponding to a more advanced stage of bud or leaf development (0 means bud stage and 6—fully developed leaf) (Table 1). Growth cessation was estimated by recording autumn leaf coloring stage. Again, values from 1 to 5 were assigned, with 5 corresponding to the stage when all leaves were lost. 

### 2.2. Sample Collection

The raw material needed for research was collected using a cordless drill in July of 2020. The top layers of bark were collected. Five trees from each half-sib family represent five biological replicates. In total, samples studied were: 2 populations × 5 half-sib families × 5 biological replicates. The collected plant material was dried at 40 °C for 24 h before use in testing. A detailed description is shown Table 2.

### 2.3. Extract Preparation

Extracts were prepared from 0.5 g of air-dried bark samples homogenized using an A11 basic analytical mill (Laboratory Equipment, Staufen, Germany), which was shaken with 10 mL of either 75% methanol (MeOH) or 10 mL of distilled water (dH_2_O) for 24 h at room temperature using a Kuhner Shaker X electronic shaker (Adolf Kühner AG, Birsfelden, Switzerland). The obtained extracts were filtered through Whatman no. 1 filter paper, with a retention of 5–8 µm.

### 2.4. Quantification of Total Phenol Content

Total phenol content (TPC) was determined using Folin-Ciocalteu reagent according to Slinkard and Singleton’s method [42]. The reaction mixture used in this study is detailed in Table 3. The absorbance was measured using Synergy HT Multi-Mode Microplate Reader (BioTek Instruments, Inc., Bad Friedrichshall, Germany) at 760 nm against the reagent blank (MeOH for methanol extracts and dH_2_O for aqueous extracts). The phenol content was expressed as chlorogenic acid per gram of weight of bark (mg CAE/g). The standard calibration curve equation used for MeOH samples: y = 5.5358x − 0.0423 (R^2^ = 0.9975); for dH_2_O samples: y = 5.5x − 0.0451 (R^2^ = 0.999). 

### 2.5. Quantification of Total Flavonoid Content

The total flavonoid content (TFC) in the extracts was determined according to a method described in Lučinskaitė et al. [43]. The reaction mixture used in this study is detailed in Table 3. The absorbance of the mixture was recorded at 470 nm on the Synergy HT Multi-Mode Microplate Reader. The same blanks (MeOH and dH_2_O) as those used for TPC were used here as well. The flavonoid content was expressed in milligrams of catechin per gram of weight of bark (mg CE/g). The standard calibration curve equation for MeOH samples: y = 11.616x + 0.0634 (R^2^ = 0.9983); for dH_2_O samples: y = 10.201x + 0.0527 (R^2^ = 0.9983).

### 2.6. Quantification of Antioxidant Activity

#### 2.6.1. DPPH (2,2-Diphenyl-1-picryl-hydrazyl-hydrate)

Total free radical scavenging capacity of the extracts from different *Q. robur* samples were estimated according to Ragaee et al.’s [44] method. The reaction mixture used in this study is detailed in Table 4. Absorbance was measured at 515 nm using Genesys 6 spectrophotometer (Thermo Spectronic, Waltham, MA, USA) against an equal amount of DPPH and 75% methanol as a blank (or water and DPPH in the case of dH_2_O extracts). The standard calibration curve equation was y = 0.2074x − 0.004 (R^2^ = 0.9907). The radical scavenging activity was calculated as antioxidant Trolox equivalents per gram of sample and calculated to Equation (1):

(1) TE=(c×V)/m 

where *c* =Trolox concentration (mM/mL); *V* = the extract volume (mL); *m* = the sample amount (g).

#### 2.6.2. ABTS (2,2′-Azino-bis(3-ethylbenzothiazoline-6-sulfonic acid)

Free radical scavenging activity in plant extracts was determined by ABTS radical cation decolorization assay [43]. The reaction mixture used in this study is detailed in Table 4. After 16 h, the mixture was diluted with dH_2_O until it reached 0.700 ± 0.2 absorbance (734 nm). dH_2_O was used as a blank. Absorbance was measured at 734 nm using Genesys 6 spectrophotometer (Thermo Spectronic) (ABTS and 75% methanol as a blank or water and ABTS in the case of dH_2_O extracts). Trolox was used as the standard. Twenty-five milligrams of Trolox (97%, Sigma-Aldrich, St. Louis, MO, USA) was dissolved in 75% MeOH (LaboChema, Vilnius, Lithuania). The stock standard of Trolox was 1 mg/mL, and 1, 2, 3, 4, or 5 mL of stock standard was used, diluted with 10 mL of 80% (*v*/*v*) ethanol, to determine the effect of varying the Trolox concentration. The standard calibration curve equation was y = 0.2734x + 0.0304 (R^2^ = 0.9842). The radical scavenging activity was calculated as antioxidant Trolox equivalent per gram of sample and calculated to Equation (1).

### 2.7. Statistical Analysis

Group means and standard errors were calculated using Microsoft Excel. Statistical data analysis was performed using the SPSS program (IBM, version 28.0.1.1.). The Kruskal–Wallis H test was used for analysis as a non-parametric alternative to one-way ANOVA. During this test, differences are determined by comparing the mean ranks of groups. A post hoc Dunn’s test was performed to indicate differences between individual pairs [32,45,46].

The importance or random and fixed effects on variance were analyzed using SAS software (SAS Institute Inc. 2002–2012, version 9.4). SAS UNIVARIATE procedure was used to check if residuals follow normal distribution. Data on TFC in dH_2_O extracts showed significant deviation from the normal distribution, thus logarithmic transformation was applied to get appropriate normality. Test for homogeneity of trait variance was done with GLM procedure Levene’s Test. Tukey’s studentized range (HSD) test in GLM procedure was used to carry out multiple comparisons between traits.

The variance components were calculated using the SAS MIXED procedure (REML method):

(2) $$Y_{ijkl}=µ+R_{i}+P_{j}+F_{k}+E_{ijkl}$$

where µ is the grand mean, *R_i_* is the fixed effect of replicate *i*, *P_j_* is the random effect of population *j*, *F_k_* is the random effect of family *k*, and *E_ijkl_* is the residual error. Standard errors of the estimates of variance components were calculated by Taylor expansions and the asymptotic covariance matrix of the estimates was obtained from MIXED procedure [47].

## 3. Results

Based on bud burst phenology evaluation it was concluded that Josvainiai (Jox) population is of early bud burst phenology and Dūkštos (D) population is of the late bud burst phenology.

### 3.1. Total Phenol (TPC) and Total Flavonoid (TFC) Content

**Figure 1:**
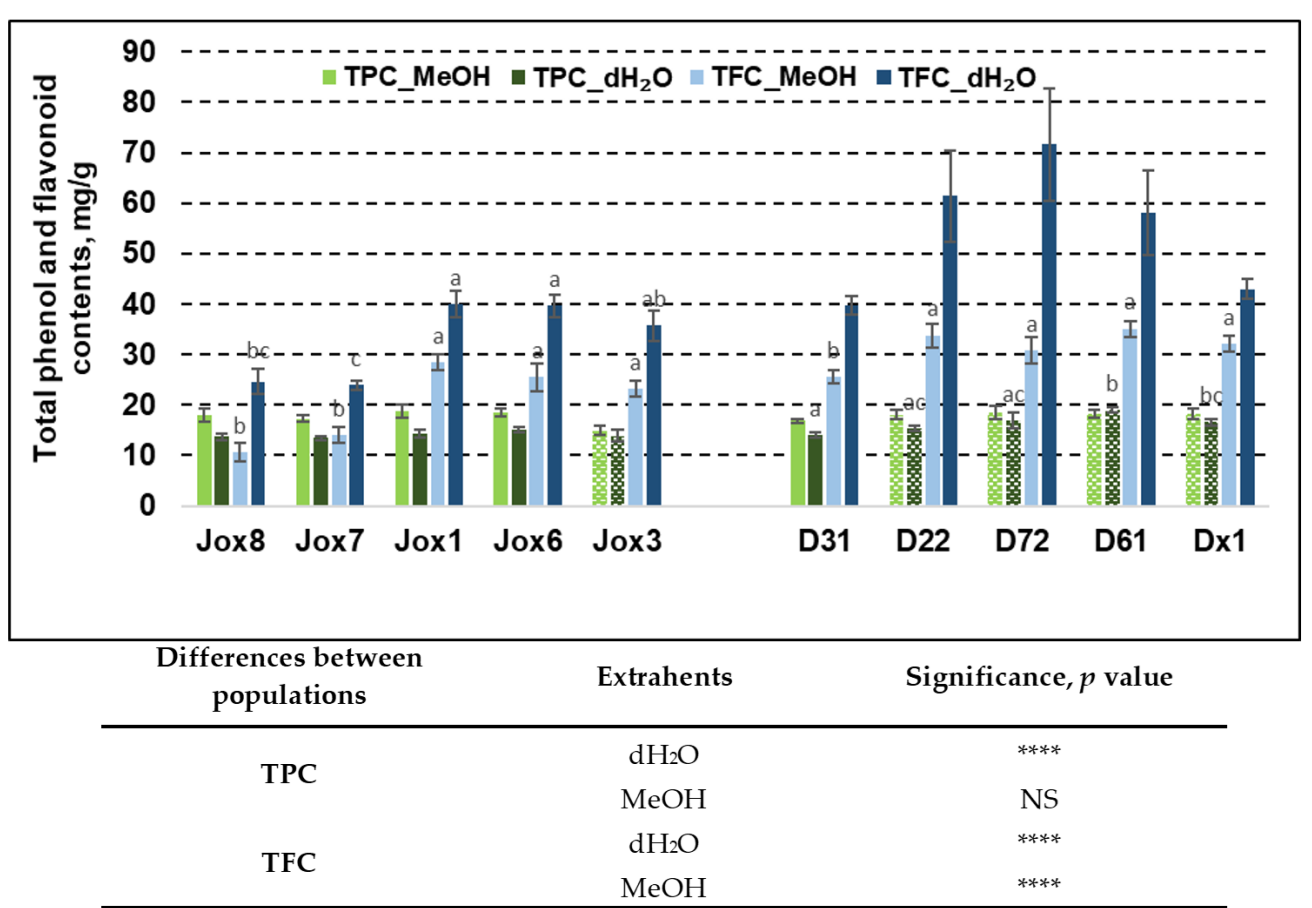
Means (mM/g fresh weight) ± SE of total phenol (TPC) and total flavonoid (TFC) content in 10 *Q. robur* families. Significance was calculated using the Kruskal–Wallis H test for ranks and post hoc Dunn’s test for pairs (*p* < 0.05). Different letters next to the same colors indicate significant differences between families, solid colors indicate significant differences between extrahents and differences among the studied populations are noted in the table (*p* values: ****—≤0.0001, NS—not significant). Different populations are indicated by group designations Jox and D.

It was shown that secondary metabolite (TPC and TFC) content in the oak bark varied significantly between populations, half-sib families, and when using different extrahents (Figure 1).

Our results showed that TPC in both populations (Josvainiai and Dūkštos) was similar (from 13.71 mg/g to 18.71 mg/g). The highest amount of TPC was observed in Josvainiai population, when the extrahent was MeOH—18.71 mg/g (Jox1 family). Bark samples from Dūkštos population family D61 had similar amount of TPC—18.93 mg/g. In Jox (Josvainiai) population TPC variation between half-sib families was not significant, irrespective of extrahent used. Furthermore, in Jox (Josvainiai) population TFC varied significantly between families, irrespective of extrahent used. In Dūkštos (D) population, significant differences between families were noted in TPC extracted with water and TFC extracted with methanol.

It was noted that late budburst population (Dūkštos, D) had higher amount of TFC, compared to Josvaniai (Jox) population, irrespective of extrahents used. The highest amount of TFC in the oak bark samples was found in Dūkštos population, where dH_2_O was used for extraction—71.61 mg/g—family D72. Meanwhile, the highest amount of TFC in Josvainiai (Jox) population, the same as TPC, was observed in the Jox1 family—39.95 mg/g. In addition, our study showed that TPC variation within individuals from the same half-sib family using different extrahents was significant. For example, it was determined that Josvainiai (Jox) population exhibited significant TPC variation in four out of five tested families. When looking at significant differences between extrahents, TPC extraction using methanol was more effective. It was noted that TPC accumulation in Dūkštos (D) population was significantly different between extrahents only in one family (D31). TFC variation using different extrahents was significantly different in both populations and in all families. In all cases, better TFC values were achieved using water as an extrahent rather than methanol. The highest difference between extrahents in Josvainiai (Jox) population was observed in family Jox6. Meanwhile, the highest difference between extrahents in Dūkštos (D) population was noted in family D72.

Statistical analysis showed that differences between two populations (as they represent oak phenology) also were significant in most cases. As shown in Figure 1, TFC differences between populations were more noticeable, compared to TPC variation. When using water as an extrahent, the amount of phenols and flavonoids in both populations were statistically different. Meanwhile, when using methanol, the difference was only noted in TFC.

Analysis of random and fixed effects on variance showed that genotype (half-sib family) had a significant effect on TFC extraction by methanol and by water, while tree phenology had no significant effect. This can only partly be explained by within-group variation as can be seen from replicate significance levels. At the same time, TPC was unaffected by either oak tree phenology or genotype (Table 5).

### 3.2. Radical Scavenging Activity

**Figure 2:**
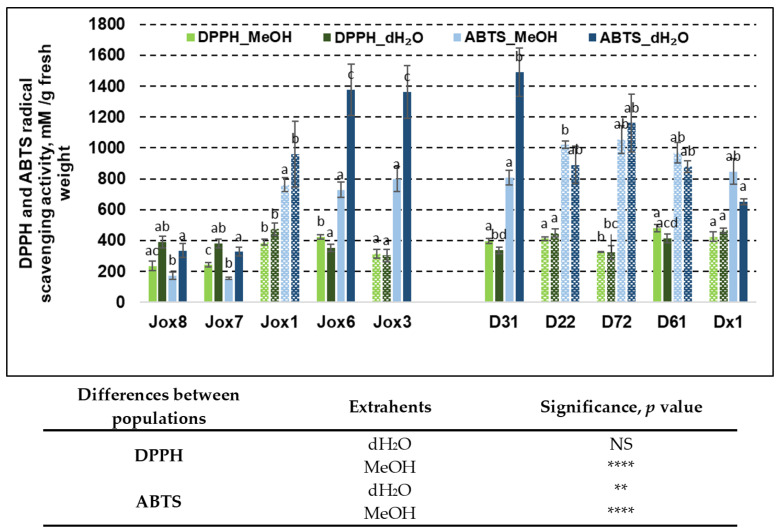
Means (mM/g fresh weight) ± SE of DPPH and ABTS scavenging activity in 10 *Q. robur* families. Significance was calculated using the Kruskal–Wallis H test for ranks and post hoc Dunn’s test for pairs (*p* < 0.05). Different letters next to the same colors indicate significant differences between families, solid colors indicate significant differences between extrahents, and differences among the studied population are noted in the table (*p* values: **—≤0.01, ****—≤0.0001, NS—not significant). Different populations are indicated by group designations Jox and D.

As with phenolics, our results show that oak population, sample extraction method and half-sib family had significant impact on free radical scavenging activity (Figure 2).

Antioxidant activity in oak bark was evaluated by DPPH and ABTS radical scavenging assays. Radical scavenging activity varied between 235.28 mM/g and 481.33 mM/g (DPPH method) and from 172.03 mM/g to 1376.22 mM/g (ABTS method) in Jox (Josvainiai) population. In late budburst population (Dūkštos) antioxidant activity varied between 337.50 mM/g and 481.33 mM/g (DPPH analysis) and from 652.23 mM/g to 1490.17 mM/g (ABTS analysis), showing significant variation in both populations between assays and within the results from either assay. Analysis showed that radical scavenging activity as measured by the ABTS method had more obvious differences between populations, compared to the DPPH assay.

Significant differences between half-sib families in the same population were observed in both assays and when using both extrahents. DPPH assay resulted in significant differences between extrahents in five families. In three families, better results were achieved when using methanol, in the other two—when using water. Family Jox1 exhibited highest antioxidant capacity in Josvainiai population, while family D61 was the best in Dūkštos population. On the other hand, antioxidant activity when using ABTS assay was significantly different in six families. In five of them, higher values were achieved when using water. In three families, Jox3, Jox6, and D31, radical scavenging activity as measured by ABTS assay, were especially high.

Results showed that differences between two populations (as they represent oak phenology) also were significant in most cases, similarly as with TPC and TFC. Except in the case of radical scavenging activity as shown in Figure 2, differences between populations in antioxidant activity measured by DPPH assay were not as big and in the results of ABTS assay. When using water as an extrahent, the free radical scavenging activity as measured by DPPH assay was not significantly different between populations, but it was different when using methanol as an extrahent. ABTS assay resulted in significant differences between populations regardless of extrahent used.

Furthermore, analysis of random and fixed effects on variance showed that genotype (half-sib family) had a significant effect on DPPH extraction by both means. Tree phenology had no significant effect on this. ABTS extraction by methanol was also affected by genotype, but not phenology. In addition, neither genotype nor tree phenology affected ABTS extraction by water (Table 5). All in all, ~96% of all variance in methanol extracts was determined by tree genotype (TPC, TFC, ABTS, DPPH). Methanol extracts were also less affected by replicate variation. Moreover, only around 35% of variance was determined by genotype in water extraction for all 4 tests. Water extraction was also more affected by replicate variation.

## 4. Discussion

As noted previously, multiple factors can determine the amounts of bioactive compounds a plant produces at one time. It has been shown that different species of oak synthesize different amounts of tested compounds, such as *Q. alba*, *Q. robur,* and *Q. petraea* as noted by Cabrita et al. [48] and Jordao et al. [4]. Phenolic acids, aldehydes and furanic derivatives were tested by Cabrita et al. Among them was the previously discussed ellagic acid [48]. Fernández de Simón et al. observed that the same wine aged in barrels of *Q. robur* and *Q. petraea* barrels (same species trees from different origin sites) for 21 months exhibited statistically significant differences in terms of ellagic acid and trans-resveratrol [49]. It is noteworthy that ellagitannins, precursors of ellagic acid, were found to be largely responsible for the antioxidant activity of oak wood [50]. Additionally, Prida and Puech showed that *Q. robur* grown in France and Eastern Europe diverged on the basis of their biochemistry, of which several chemicals were predominantly responsible, i.e., eugenol, 2-phenylethanol, vanillin, vescalagin, cis/trans-whiskey lactone ratio, and roburin B. In the same study, the authors also noted the vast differences between oak species [51]. Moreover, Kovalikova et al. also reported on this issue. She noted that *Q. robur* from different locations have different amounts of soluble phenols and flavonoids [35]. Another study on 30 *Q. robur* full sibs showed that phenols, flavonoids, tannins, and lignins were not affected by tree genotype all that much (up to 10% ± SE of total variation) [52]. Our own work with other tree species has shown that even when grown in carefully monitored and unified in vitro conditions two different genotypes of *Populus* spp. can produce different amounts of phenols, flavonoids, and photosynthesis pigments [31]. Furthermore, we observed the same tendencies in ash, spruce, and pine trees as well as blueberries and lingonberries [32,43,47,53,54]. All these examples align perfectly with the findings of this study, whereby we observed statistically significant differences in the amounts of TPC, TFC and antioxidant scavenging capacity diverging in different half-sib families grown in the same site and belonging to the same population.

In this work we also looked into the use of two extrahents—methanol and water. Results show that all four tests exhibited statistically significant differences in the amount of phenols, flavonoids, and antioxidants extracted. Similar results were noted in a review by Ignat et al. Their analysis of multiple works showed that methanol extraction is oftentimes more effective, but is less commonly used in food industries due to methanol toxicity [55]. Lavado et al. tested how different extracts of cork oak behaved and observed that water:ethanol extracts exhibited higher antioxidant activity than using just water or just ethanol. These mixed extrahents allowed for greater amounts of phenols, flavonoids, and condensed tannins, but not tocopherols. In the case of the latter, pure ethanol extrahents worked best [7]. Similarly, Šukele et al. presents results on using different extrahents on oak bark. They report acetone and 30% ethanol having the best outcome in terms of using these extracts for growth control of *Streptococcus agalactiae*, *Streptococcus uberis*, *Serratia liquefaciens*, and *Staphylococcus aureus* [18]. In a 2018 article Valencia-Aviles et al. report that hot water extracts were more effective that using 90% ethanol for both phenols and antioxidants [37]. Furthermore, Arina and Harisun observed that even using the same extrahent, different extraction temperatures still significantly affected the outcome [56]. All in all, these results collectively show that it is important to see which extraction methodology and which extrahent is more appropriate to use in any given case.

The most unique aspect of our investigation was the determination of whether *Q. robur* phenology can be a determining factor in the amount of bioactive compounds a tree produces. This is different from the effect tree genotype has, at tree phenotype can be determined both by genotype and by environmental conditions [57,58]. Previously, this characteristic has been shown to impact enzymatic activities in oak symbiotic fungus *Lactarius quietus*. Specifically, enzymes that contribute to the degradation and mobilization of carbon-rich components of the dead plant [59]. More importantly, it was demonstrated by Barber and Fahey that *Quercus alba* expressed differences in leaf antioxidant capacity of phenolics depending on oak phenology, i.e., early bud burst oaks had lower oxidative capacity in the first weeks of leaf growth as compared to the late bud burst oaks [60]. In our study, we observed significant differences in TPC (just water), TFC (both extrahents), DPPH (just methanol), and ABTS (both extrahents) parameters between both populations. Furthermore, it could be said that overall Josvainiai population (early bud burst) did produce less phenolics and expressed lower antioxidant capacity than Dūkštos population (late bud burst). This is a directly comparable result to that of Barber and Fahey, but it is worth noting they worked with a different oak species, and as was noted before, different oak species diverge in bioactive compound production.

## 5. Conclusions

The amount of total phenols, total flavonoids, and the antioxidant scavenging activity as expressed by ABTS and DPPH assays are different between the bark of *Quercus robur* genotypes (half-sib families), oaks with different phonologies (early or late bud burst populations), and different extrahents (75% methanol and distilled water). Overall, late bud burst population exhibited higher values in all parameters measured. Thus, in order to optimize extraction of desired bioactive compounds of phenolic origin from *Q. robur* bark, it is pertinent to take these factors into account. We would also like to emphasize that oak bark has a huge potential to be used as a natural product in supplement, additive, and other industries.

## Figures and Tables

**Table 1:** The budburst stages of half-sib families of pedunculate oak (arranged from earliest (Josvainiai, Jox) to latest (Dūkštos, D)).

Josvainiai Oak Population	Budburst Stage	Standart Deviation	Dūkštos Oak Population	Budburst Stage	Standart Deviation
Jox8	5.4	0.5	D31	3.2	1.3
Jox7	5.0	0.6	D22	2.8	1.4
Jox1	4.8	0.5	D72	2.8	1.4
Jox6	4.0	0.9	D61	2.2	1.6
Jox3	3.6	1.0	Dx1	2.1	1.3

**Table 2:** Used variables for the evaluation of biological compounds and antioxidant activity changes in oak bark: different populations, different half-sib families, and sample extraction method.

Population		Half-Sib Families (Genotype)		Extrahent	
Early burst phenology: Josvainiai (Jox)	Late burst phenology: Dūkštos (D)	Josvainiai (Jox): Jox8 Jox7 Jox1 Jox6 Jox3	Dūkštos (D): D31 D22 D72 D61 Dx1	MeOH (75% in water)	dH_2_O

**Table 3:** Reaction mixture used to evaluate total phenolic content (TPC) and total flavonoid content (TFC).

Total Phenolic Content (TPC)	Total Flavonoid Content (TFC)
Reaction mixture: 100 µL sample + 2500 µL dH_2_O + 100 µL *Folin–Ciocalteu* reagent (2 N) (wait 6 min) + 5000 µL Na_2_CO_3_ (25%, *w*/*v*). The mixture was left for 30 min at room temperature.	Reaction mixture: 1000 µL sample + 300 µL NaNO_2_ (5%, *w*/*v*) (wait 5 min) + 500 µL AlCl_3_ (2%, *w*/*v*) (wait 6 min) + 500 µL NaOH (1M).

**Table 4:** Reaction mixtures used to evaluate antioxidant activity with two different methods (DPPH and ABTS).

Radical Scavenging Activity	
DPPH Method	ABTS Method
Reaction mixture: 100 µL samples + 400 µL MeOH (75%) + 1000 µL DPPH solution (0.1 mM). Mixture was incubated at room temperature in the dark for 16 min. DPPH solution preparation: 11.8 mg was dissolved in 300 mL MeOH (100%).	Reaction mixture: 50 µL samples + 2000 µL ABTS solution. Mixture was incubated at room temperature in the dark for 10 min. ABTS solution preparation: 56 mg of ABTS (>99%, Fluka, Buchs, Germany) was dissolved in 50 mL of dH_2_O. ABTS radical cation was prepared by reacting ABTS stock solution with 200 µL of K_2_S_2_O_8_ (70 nM). The mixture was held in the dark at room temperature for 16 h before it was used.

**Table 5:** Variance components for random effects as percent from the total variation and significance of the fixed effect. Level of significance is denoted by *: 0.05 > *p* > 0.01, **: 0.01 > *p* > 0.001, ***: *p* < 0.001, ns—not significant.

Trait	Variance Components of Random Effects, %		Significance of Fixed Effect
	Phenology (Population)	half-Sib Family (Genotype)	Replicate (Individual)
DPPH_MeOH	20.0 ± 39.1	34.5 ± 18.8 *	ns
ABTS_MeOH	42.4 ± 69.6	32.3 ± 17.0 *	ns
TPC_MeOH	0.0	3.5 ± 4.7	***
TFC_MeOH	38.1 ± 61.1	22.6 ± 12.6 *	ns
DPPH_dH_2_O	0.0	18.4 ± 11.3 *	**
ABTS_dH_2_O	0.0	41.7 ± 21.5	*
TPC_dH_2_O	17.2 ± 28.5	9.8 ± 7.3	*
TFC_dH_2_O	37.9 ± 59.2	16.7 ± 9.9 *	ns

## Data Availability

All data will be made available upon request.
